# Low Power Consumption 3D-Inverted Ridge Thermal Optical Switch of Graphene-Coated Polymer/Silica Hybrid Waveguide

**DOI:** 10.3390/mi11080783

**Published:** 2020-08-18

**Authors:** Yue Cao, Yunji Yi, Yue Yang, Baizhu Lin, Jiawen Lv, Haowen Zhao, Fei Wang, Daming Zhang

**Affiliations:** State Key Laboratory of Integrated Optoelectronics, College of Electronic Science and Engineering, Jilin University, 2699 Qianjin Street, Changchun 130012, China; yuecao19@mails.jlu.edu.cn (Y.C.); yiyj@jlu.edu.cn (Y.Y.); yangyue19@mails.jlu.edu.cn (Y.Y.); linbz19@mails.jlu.edu.cn (B.L.); lvjw18@mails.jlu.edu.cn (J.L.); zhaohw1918@mails.jlu.edu.cn (H.Z.); wang_fei@jlu.edu.cn (F.W.)

**Keywords:** graphene, three-dimensional hybrid integrated, inverted ridge thermal optical switch, side electrode structure

## Abstract

An inverted ridge 3D thermal optical (TO) switch of a graphene-coated polymer/silica hybrid waveguide is proposed. The side electrode structure is designed to reduce the mode loss induced by the graphene film and by heating the electrode. The graphene layer is designed to be located on the waveguide to assist in the conduction of heat produced by the electrode. The inverted ridge core is fabricated by etching and spin-coating processes, which can realize the flat surface waveguide. This core improves the transfer of the graphene layer and the compatibility of the fabrication processes. Because of the opposite thermal optical coefficient of polymer and silica and the high thermal conductivity of the graphene layer, the 3D hybrid TO switch with low power consumption and fast response time is obtained. Compared with the traditional TO switch without graphene film, the power consumption of the proposed TO switch is reduced by 41.43% at the wavelength of 1550 nm, width of the core layer (a) of 3 μm, and electrode distance (d) of 4 μm. The rise and fall times of the proposed TO switch are simulated to be 64.5 μs and 175 μs with a *d* of 4 μm, and *a* of 2 μm, respectively.

## 1. Introduction

Three-dimensional (3D) hybrid integrated chips have the advantages of a high integration degree of photonic chips, improvement and diversification in device function, and low signal crosstalk; consequently, these chips can be applied to optoelectronic integration fields and 3D integration fields [1,2]. A variety of optical devices such as vertical amplifiers [3,4], optical waveguides [5,6], optical switches [7,8], lasers [9,10], and optical detectors [11,12] can be integrated on a single chip according to their respective functions. The hybrid integration of different material systems is the key factor in optical devices. However, integrating different materials has the problems of refractive index mismatch, large coupling loss, and poor material compatibility. Up to now, matched materials are mainly focused on silica, glass, and polymer in 3D integration optical devices [13,14].

A key goal of 3D hybrid integrated optical devices is to realize the optical path switching between different layers. Therefore, the X-junction vertical structure optical switch has been proposed [15,16,17]. In 2018, Qian Qian Song fabricated an X-junction thermo-optic with a silicon oxynitride (SiON) core and polymer cladding, realizing a power consumption of 59.6 mW and rise and fall times of 1.42 ms and 0.85 ms, respectively [18]. Among the structures of X-junction optical switches, the organic–inorganic hybrid X-junction optical switch can realize the lower power consumption because of the different thermal optical (TO) coefficients of the polymer and inorganic material. In contrast, the organic–inorganic hybrid Mach Zehnder interferometer (MZI) optical switch can realize lower power consumption than the X-junction optical switch [19]. In 2012, a polymer/silica hybrid MZI thermo-optic switch was fabricated with a power consumption lower than 7.2 mW and rise and fall times of 106 μs and 93 μs, respectively [20]. However, it was a planar thermal optical (TO) switch.

To further reduce the power consumption of the vertical optical devices, the polymer/silica hybrid MZI TO switches with a graphene film have attracted attention. Two-dimensional graphene material has many excellent optical properties such as large thermal conductivity, high carrier mobility, and wide optical response spectrum [21]. Therefore, graphene-based TO devices can realize lower power consumption and fast response time [22,23,24]. However, this device based on graphene material generally has a large optical loss due to the optical absorption of graphene material, and the fabrication process of graphene-based 3D MZI TO devices is limited. The first reason is that the uneven surface of the waveguide destructs the graphene film and affects the performance of the switch. Moreover, the structure of multilayer integration leads to alignment problems of the template after spin-coating a new layer that is not conducive to the matching between different layers of the waveguide. To solve these problems, the inverted ridge waveguide structure can be applied, realizing the flat surface waveguide, helping transfer the graphene layer. The structure of the inverted ridge waveguide can be realized by an etching process [25,26] or imprinting processes [27] such as hot imprinting [28] and nanoimprinting [29]. Because the etching process is beneficial to realize the compatibility of silica and polymer materials, the polymer/silica hybrid device with fast response time can be realized. Moreover, for the devices with loss compensation, the silica grooves structure fabricated by the etching process can avoid the solubility of core layer and cladding materials due to the benzene in the solvent. Finally, the etching process also has the advantages of high etching precision and low loss.

In this paper, a 2 × 2 MZI-inverted ridge TO switch of a graphene–polymer–silica hybrid waveguide with side electrode structure is proposed. The waveguide grooves are designed to be fabricated by the etching process. Then, the polymer core layer was obtained by polymer spin-coating in grooves. This process can realize the compatibility of silica and polymer materials and the flat surface waveguide, thus helping in the transfer of the graphene film. Because of the high thermal conductivity of the graphene layer and the opposite thermal optical coefficient of polymer and silica, the 3D-inverted ridge TO switch of the graphene–polymer–silica hybrid waveguide with low power consumption and fast response time can be obtained.

## 2. Device Design and Theoretical Analysis

**Figure 1:**
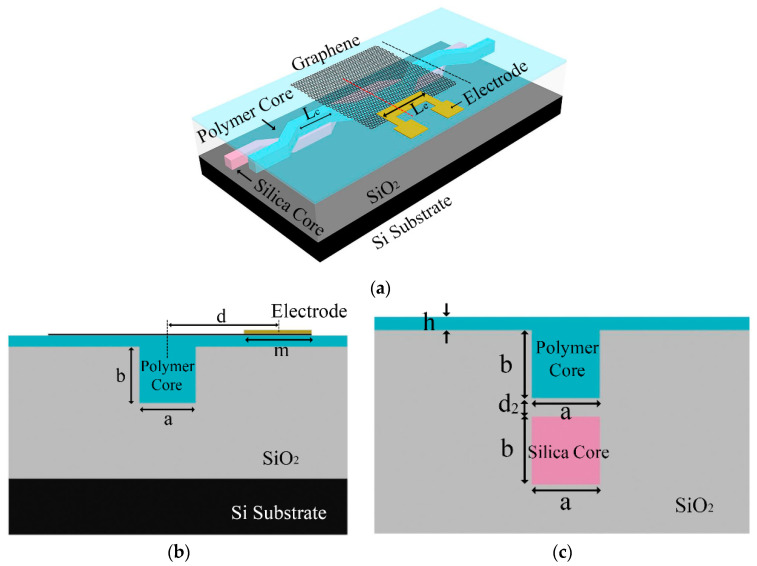
(**a**) Three-dimensional structure; (**b**) two-dimensional cross-sectional structure in the heating region (the position of the red dotted line in Figure 1a); and (**c**) two-dimensional cross-sectional structure in the 3-dB coupling regions (the position of the black dotted line in Figure 1a) of the inverted ridge 3D hybrid thermal optical (TO) switch with graphene film and side electrode structure.

The 3D structure of the MZI-inverted ridge 3D hybrid TO switch with a graphene film is shown in Figure 1a. Figure 1b,c show the cross-sectional structure in the heating region and the 3-dB coupling regions, respectively. The device consists of a silica core layer, silica cladding, polymer inverted ridge core layer, gold electrode, graphene layer, and silicon under substrate. Because of the large thermal conductivity of the graphene material coated on the polymer core layer, it helps conduct the heat generated by the electrode in the polymer waveguide, increasing the change of the effective refractive index due to the increase in the temperature in the polymer core layer.

The dimensions of the core layers are *a* = *b* = 3 μm. The parameters of *m* = 6 μm, *L_e_* = 1 cm, and *d_2_* = 0.6 μm indicate the width of the electrode, length of the electrode, and coupling gap between two waveguide core layers, respectively. The thickness of the slab layer is *h* = 1.22 μm. The coupling length of the waveguide in the vertical direction is *L_c_*. The horizontal distance between the center of the core layer and the center of the electrode is *d*. *n_c_*_1_ = 1.48, *n_c_*_2_ = 1.478, and *n*_1_ = 1.444 are the refractive indices of the silica core layer, polymer core layer, and silica cladding layer, respectively. When the effective refractive index of the two core layers is equal, the optical field can be theoretically fully coupled in a 3-dB coupler. When heating the electrode, the effective refractive index of the waveguide changed, realizing the phase change of π.

The thermo-optical coefficients of the polymer and silica are −2.36 × 10^−4^/°C and 1.28 × 10^−5^/°C. The phase change ∆Φ can be expressed as:

(1) $$\Delta \Phi =\frac{2\pi }{\lambda }\Delta N_{\mathrm{neff}}\times L$$

where the ∆N_neff_ is the effective refractive index change, and *λ* is the wavelength of waveguide (set at 1550 nm). Because of the large thermal conductivity of a graphene layer (thermal conductivity of graphene layer *k_g_* = 5300 W/mK, thermal conductivity of polymer *k_p_* = 0.19 W/mK), the graphene layer is coated on the waveguide to assist the conduction of heat produced by the electrode. This process helps realize the phase shift at a lower power consumption (*P*) of the 3D hybrid TO switch.

**Figure 2:**
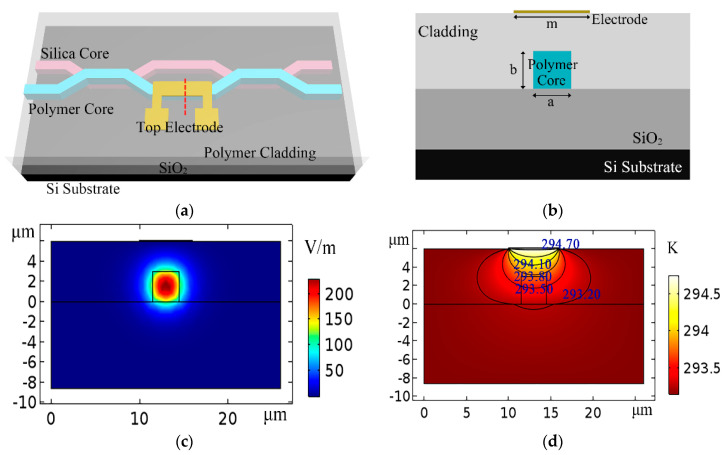
TO switch with top electrode structure by a lithography process. (**a**) Three-dimensional structure; (**b**) two-dimensional cross-sectional structure in the heating region (the position of the red dotted line in Figure 2a); (**c**) optical field distribution in the heating region; (**d**) thermal field distribution in the heating region.

**Figure 3:**
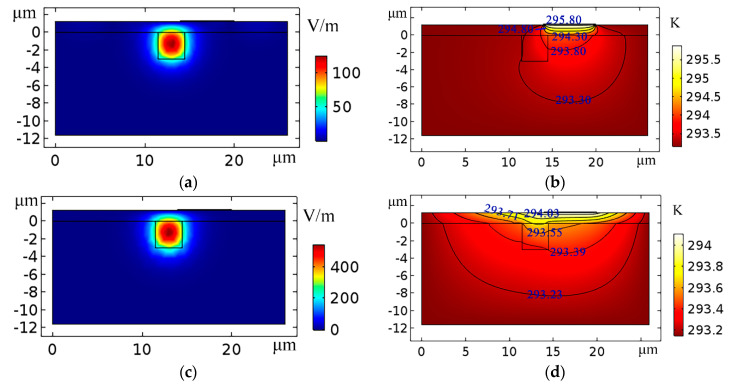
Inverted ridge TO switch with side electrode structure. (**a**) Optical field distribution in the heating region without a graphene film; (**b**) thermal field distribution in the heating region without a graphene film; (**c**) optical field distribution in the heating region for a graphene TO switch; (**d**) thermal field distribution in the heating region for a graphene TO switch.

The optical field distribution and the thermal field distribution of the devices are simulated by finite element analysis software. The simulation processes include geometry build, add material, physics analysis, meshing build, study, and results analysis. For the optical field analysis, the physics interfaces are “electromagnetic waves” and “frequency domain”. The meshing method and the study method are “physics-controlled mesh” and “mode analysis”, respectively. For the thermal field analysis, the physics interface is “heat transfer in solids”. The meshing method and the study method are “free triangular mesh” and “stationary”, respectively. First, the top electrode structure of the TO switch by lithography process has been simulated (as shown in Figure 2). *n_2_* is the refractive index of the polymer cladding layer. To realize the complete coupling of light field, the structure of the waveguide has been optimized (*a* = *b* = 3 μm; the height of cladding *h*_cladding_ = 6 μm; *n_c_*_1_ = 1.48; *n_c_*_2_ = 1.478; *n*_1_ = 1.444; *n*_2_ = 1.451). Figure 2a,b show the 3D structure and the cross-sectional structure in the heating region, respectively, for the TO switch with top electrode structure. Figure 2c,d show the light field distribution and thermal field distribution of the TO switch, respectively. The power consumption of the TO switch with top electrode structure is 4.2 mW when the temperature change is 1.6 K. Second, the inverted ridge 3D hybrid TO switches with side electrode structure are simulated in Figure 3 (*a* = *b* = 3 μm; *n_c1_* = 1.48; *n_c_*_2_ = 1.478; *n*_1_ =1.444; *h* =1.22 μm; *d* = 4 μm). Figure 3a,b show the light field distribution and thermal field distribution of the inverted ridge TO switch without a graphene film. The temperature change is 2.74 K, and the power consumption is 7.02 mW. Figure 3c,d show the light field distribution and thermal field distribution of the inverted ridge TO switch with a graphene-assisted heating layer. The temperature change is 0.96 K, and the power consumption is 2.46 mW. Because of the large thermal conductivity of the graphene layer, the heat produced by the electrode conducts along the graphene film, increasing the temperature of the waveguide (as shown in Figure 3b,d). Because the top electrode structure requires a polymer cladding to reduce the device losses, and the 2D graphene layer helps the heat conduct in a transverse direction for the side electrode structure. Compared with the TO switch with a top electrode structure, the power consumption of the graphene-based inverted ridge TO switch with side electrode structure is reduced by 41.43% (as shown in Figure 2d and Figure 3d).

**Figure 4:**
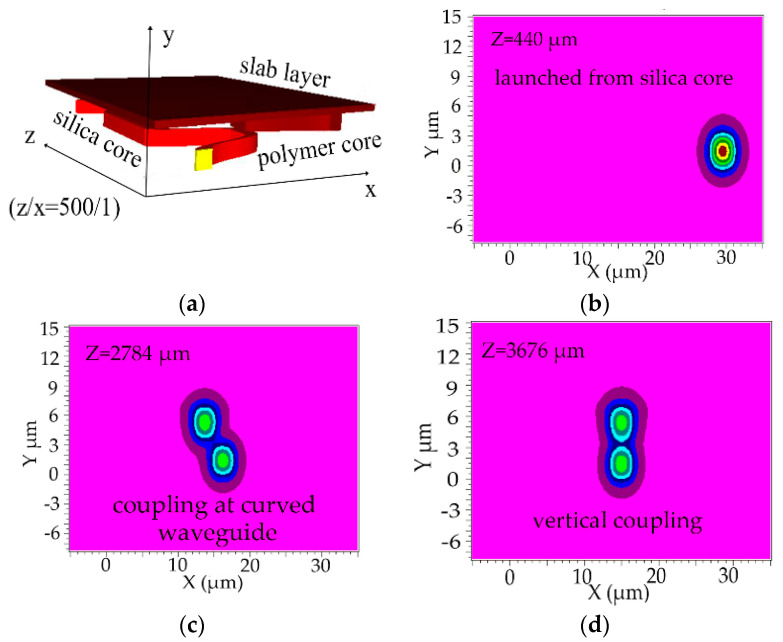

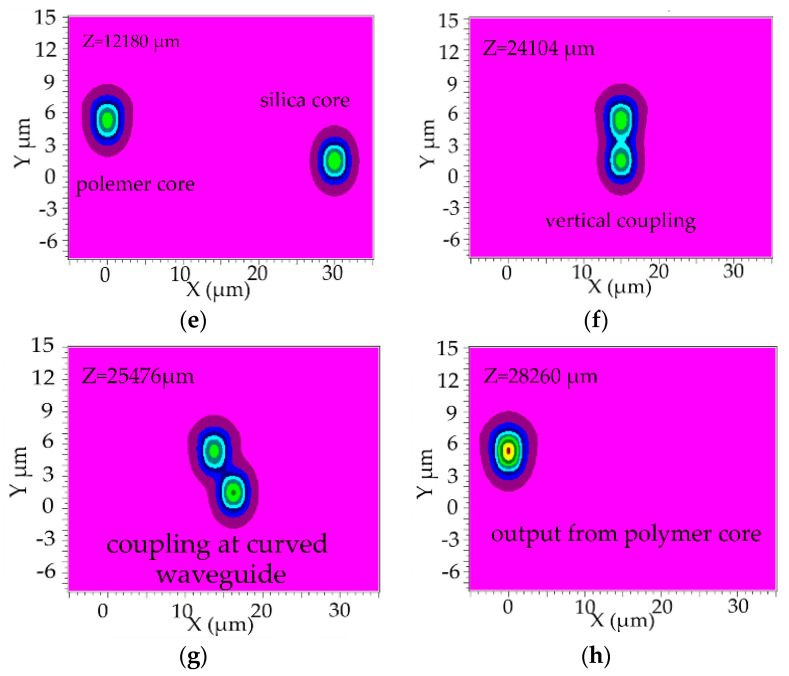
(**a**) The 3D geometric model of the inverted ridge TO switch; (**b**–**h**) corresponding to the light transmission in x–y direction at z axis positions of 440 μm, 2784 μm, 3676 μm, 12,180 μm, 24,104 μm, 25,476 μm, 28,260 μm, respectively (*a* = 3 μm, *h* = 1.22 μm; *L_c_* = 2.049 mm; *m* = 6 μm, *L* = 1 cm, *n_c_*_1_ = 1.48, *n_c_*_2_ = 1.478 and *n*_1_ = 1.444).

**Figure 5:**
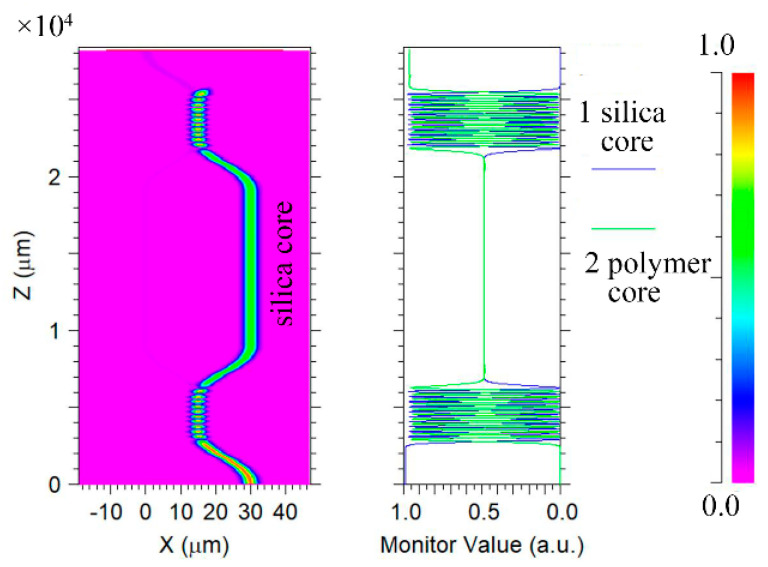
Simulated light field coupling is launched from silica core for the inverted TO switch with a graphene film and side electrode structure.

The light field coupling for the inverted ridge TO switch was simulated by 3D BeamPROP module of the Rsoft software. The light outputs from polymer core were launched from the silica core with an a of 3 μm, *h* of 1.22 μm, *L_c_* of 2.049 mm, *m* of 6 μm, *L_e_* of 1 cm, *n_c_*_1_ of 1.48, *n_c_*_2_ of 1.478, and *n*_1_ of 1.444. The 3D geometric model of the TO switch constructed with Rsoft software is shown in Figure 4a. The light transmission in x–y direction of the TO switch at z axis positions of 440 μm, 2784 μm, 3676 μm, 12,180 μm, 24,104 μm, 25,476 μm, 28,260 μm is shown in Figure 4b–h, respectively. Figure 5 shows the simulated light field coupling between silica waveguide and polymer waveguide in x–z direction. It shows that the coupling in the vertical direction can be realized in the MZI TO switch. The calculated coupling efficiency is 97.5%.

## 3. Optimization and Discussions

The modal losses of the graphene TO switch are related to the graphene film, position of the gold electrode, and dimension of the waveguide. The refractive indexes of the gold electrode and graphene film are 0.559 + 11.5i and 2.973 + 2.79i, respectively [16]. The calculation of the mode losses is dependent on the imaginary part of the effective refractive index of the waveguide, which can be expressed as [30,31]:

(2) α=10log10{exp[2×(2πλ)Im(Nneff)]}=8.68×2πλIm(Nneff)

**Figure 6:**
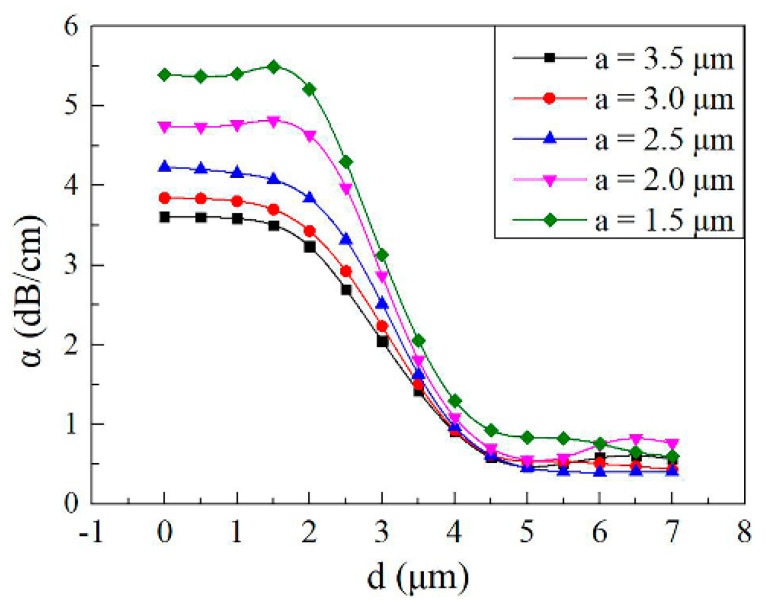
Calculated mode losses induced by graphene film and electrode with different horizontal distances between the center of the core layer and the electrode (*a*_1_ = 3.5 μm, *h*_1_ = 1.24 μm; *a*_2_ = 3 μm, *h*_2_ = 1.22 μm; *a*_3_ = 2.5 μm, *h*_3_ = 1.20 μm; *a*_4_ = 2 μm, *h*_4_ = 1.18 μm; *a*_5_ = 1.5 μm, *h*_5_ = 1.14 μm; *m* = 6 μm, *L* = 1 cm, *n_c_*_1_ = 1.48, *n_c_*_2_ = 1.478 and *n*_1_ = 1.444).

Figure 6 shows the calculated mode losses (*α*) induced by graphene film and electrode as a function of horizontal distances (*d*) between the center of the core layer and the electrode with the electrode width (*a*) from 1.5 to 3.5 μm. Five different polymer core layer dimensions are designed by COMSOL, realizing the complete coupling between two waveguide core layers. Because of the absorption of light by the side electrode, as shown in Figure 6, the mode losses are significantly reduced (less than 1.5 dB/cm) and insensitive to the width of the core layer for the side electrode structure (*d* > 4 μm).

**Figure 7:**
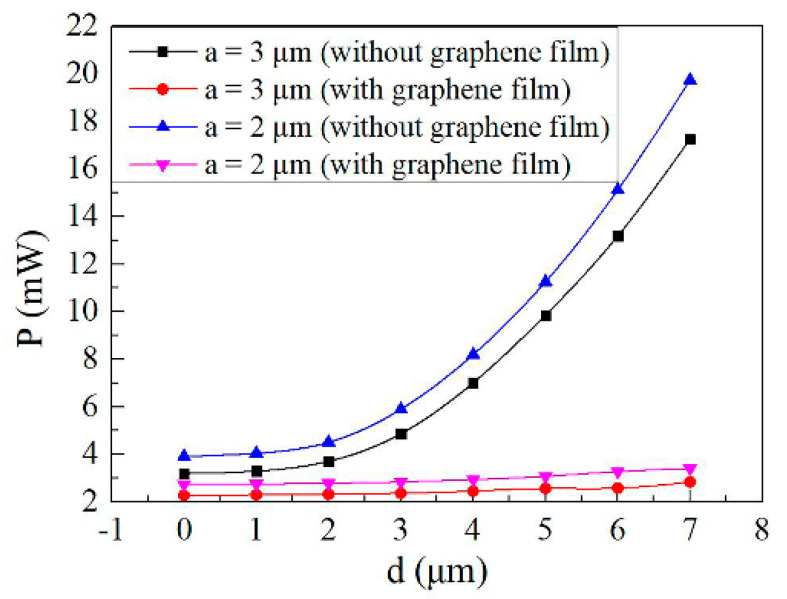
Calculated power consumption as a function of the horizontal distance between the center of the core layer and the electrode for *a* = 3 μm and *a* = 2 μm, respectively.

Figure 7 shows the calculated power consumption using finite element analysis software as a function of the horizontal distance between the center of the core layer and the electrode for *a* = 3 μm and *a* = 2 μm, respectively. For the TO switch without a graphene film structure, the power consumption significantly increases as a function of electrode distance (*d*). The power consumption is 3.2 mW with a d of 0 μm, while the power consumption increases to 17.26 mW with a d of 7 μm. For the graphene TO switch structure, the power consumption is insensitive to the electrode distance because of the large thermal conductivity of graphene film, relaxing the tolerances during the fabrication work of the devices. The results show that the power consumption for the graphene TO switch with *d* of 4 μm is reduced by 23.13% compared with the top electrode TO switch structure (*d* = 0 μm) without graphene film.

**Figure 8:**
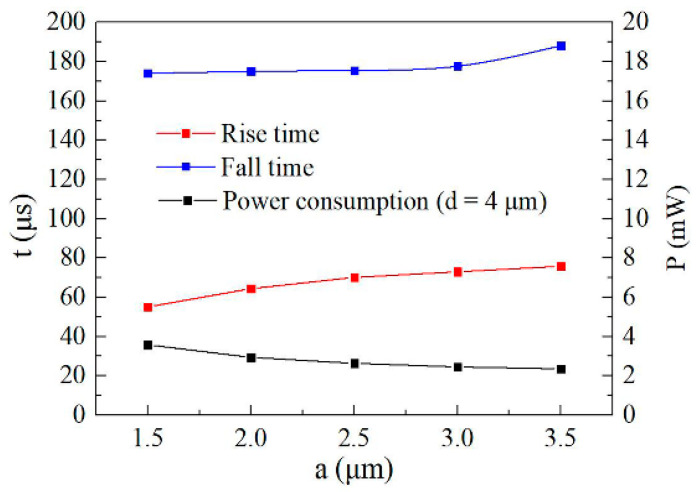
Calculated response time and the power consumption as a function of the electrode width (*d* = 4 μm).

The calculated response time and the power consumption with different electrode widths (*d* = 4 μm) are shown in Figure 8. Figure 8 shows that by reducing the width of the core layer from 4.5 to 1.5 μm, the rise time was shortened from 75.8 to 55.1 μs, the fall time was shortened from 188.2 to 174 μs, and the power consumption was increased from 2.36 mW to 3.59 mW. It indicated that the small size of the core layer can achieve a fast response time. The large size of the core layer can realize low power consumption due to the small temperature change when phase change of π was realized.

**Figure 9:**
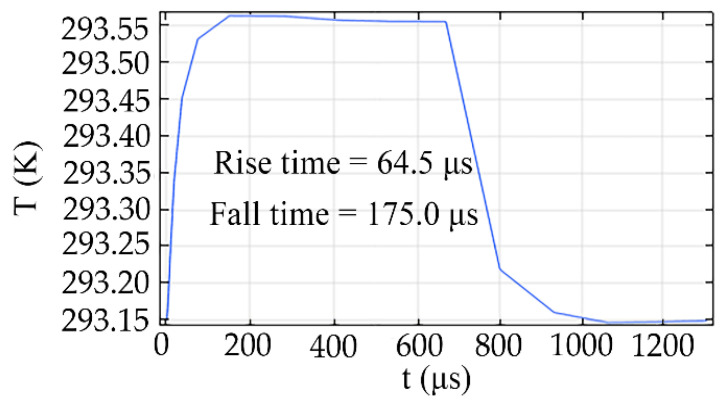
The response time curves of the graphene TO switch with a *d* of 4 μm and *a* of 2 μm.

Figure 9 shows the response time curves of the side electrode TO switch with a graphene heating layer, which is simulated by finite element analysis software. The study method is “time dependent”, and a square wave with upper limit of 0.0007 is added in the TO switch. The rise and fall times were 64.5 μs and 175 μs with a *d* of 4 μm, *a* of 2 μm, ∆*T* of 1.15 K, and *P* of 2.95 mW, respectively.

## 4. Conclusions

In summary, we have presented an inverted ridge 3D hybrid TO switch with graphene film. Because of the opposite thermal optical coefficients of polymer and silica and the high thermal conductivity of a graphene layer, a graphene-based side electrode TO switch with low power consumption and fast response time has been obtained. Compared with the traditional 3D polymer–silica hybrid TO switch with top electrode structure, the power consumption was reduced by 41.43% for the graphene TO switch with a *d* of 4 μm, *a* of 3 μm, electrode length of 1 cm, and wavelength of 1550 nm. Compared with the inverted ridge TO switch with a top electrode structure, the power consumption was reduced by 23.13% for the graphene TO switch with a d of 4 μm. Moreover, the rise and fall times were 64.5 μs and 175 with a *d* of 4 μm, *a* of 2 μm, ∆*T* of 1.15 K, and *P* of 2.95 mW, respectively.
